# Customized Pre-Epiglottic Baton Plate—A Practical Guide for Successful, Patient-Specific, Noninvasive Treatment of Neonates With Robin Sequence

**DOI:** 10.1177/1055665620972288

**Published:** 2020-11-11

**Authors:** Gül Schmidt, Anke Hirschfelder, Max Heiland, Carsten Matuschek

**Affiliations:** 1 Department of Oral and Maxillofacial Surgery, 14903 Charité—Universitätsmedizin Berlin, Corporate Member of Freie Universität Berlin, Humboldt-Universität zu Berlin and Berlin Institute of Health, Germany; 2 Department of Phoniatrics & Pedaudiology, 14903 Charité—Universitätsmedizin Berlin, Corporate Member of Freie Universität Berlin, Humboldt-Universität zu Berlin and Berlin Institute of Health, Germany

**Keywords:** pre-epiglottic baton plate, Robin sequence, upper airway obstruction, laryngomalacia, cleft palate, PEBP, RS, PRS

## Abstract

**Objective:**

Despite its efficiency and benefits in treating patients with Robin sequence (RS), the pre-epiglottic baton plate (PEBP) is not widely used. However, its acceptance might improve with specific defined parameters for indication and proper design of the velar extension. We present our 13-year, single-center experience in treating infants with RS using PEBP, focusing on the description and insertion of an endoscopically guided PEBP design along with its complications and limitations.

**Design and Innovation:**

We recommend PEBP as primary treatment for RS, suggesting a new approach of design adjustment based on endoscopic findings of multilevel upper airway obstruction.

**Setting:**

Department of cleft lip and palate.

**Patients:**

Infants with isolated or syndromic RS, period 2010 to 2019.

**Interventions:**

Pre-epiglottic baton plate treatment, intravelar veloplasty, and hard palate closure after initial PEBP treatment.

**Results:**

We treated 132 infants (isolated RS, 111; syndromic RS, 21) with PEBP. All infants with isolated RS were discharged within an average of 8 days of PEBP therapy. For them, no tracheotomy or tongue–lip adhesion procedures were needed. Only 4 of the 20 infants discharged with a nasogastric tube needed it for >2 weeks. Intravelar veloplasty and palate closure were performed after 3 and 6 months of initiating PEBP treatment, respectively.

**Conclusions:**

Application of an orthodontic device in RS therapy has not been accepted worldwide. We hope that our learning curve and recommendations about PEBP will help the implementation of this highly effective and nonsurgical treatment option.

## Introduction

The Robin sequence (RS) is a postnatal condition, which comprises micrognathia, glossoptosis, and upper airway obstruction (UAO). It is frequently associated with cleft palate and may coincide with laryngomalacia. Appropriate therapeutic response to UAO is crucial, and the therapeutic strategies vary widely, ranging from watchful waiting to surgical interventions such as tongue–lip adhesion, mandibular distraction osteogenesis (MDO), and tracheotomy (Evans et al., 2011). However, unlike these strategies, the pre-epiglottic baton plate (PEBP) is the only noninvasive alternative that allows immediate simultaneous correction of the tongue and lower jaw positions, opening the airway physiologically and ensuring tongue mobility. Pre-epiglottic baton plate improves breathing, swallowing, phonation, and overall development (Poets et al., 2019).

**Figure 1.**
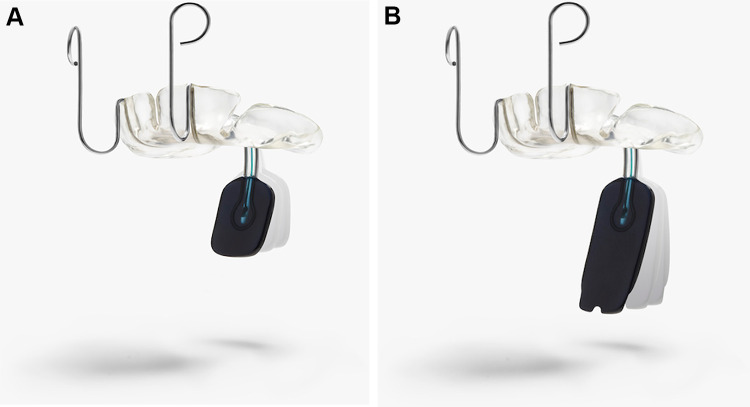
A, This figure shows the pre-epiglottic baton plate (PEBP), with the bending option in the sagittal plane. B, The differences in the length of the velar extension are observed.

Pre-epiglottic baton plate, first described by Pielou (1967), remains an unpopular therapeutic option, possibly due to the difficulties associated with the complex anatomical and functional criteria needed for the plate’s velar extension design (Figure 1A and B). Determining the location of the obstruction and the stability of the larynx are crucial for its optimal design.

*According to our observations and considering*, the multilevel character of tongue base UAO (Breugem et al., 2016), the widely used Sher classification as recommended by Müller-Hagedorn et al. (2017) provides limited details on how the velar extension should be designed, placed, and bent to avoid pressure sores and inadequate vagal stimulation. Furthermore, the velar extension should permit sufficient laryngeal elevation required for swallowing.

Inspired by the technique described by Pielou (1967) and the option to design, bend, and adjust the spur immediately at the bedside, we developed our own version of PEBP. We started using PEBP at our department in 2006. Herein, we describe our experiences and learning curve of its use from the initial years until today, which subsequently helped modify our approach. We want to introduce our treatment concept in RS, with special emphasis on the construction and adjustment of the plate and its effects on the development of patients with RS.

We like to give a detailed description of reproducible, anatomical, and technical parameters for designing PEBP. We hope that our contribution here will allow the reproducibility of our positive experiences.

## Patients and Methods

All newborns (n = 132) with clinical signs of RS, treated in our department from 2010 to 2019 were included in this study.

### Treatment Protocol for Isolated RS (n = 111)

#### Perinatal period

- Prenatal presentation of suspected RS with prenatal diagnosis and delivery in our center.
- Presentation and referral to our center after delivery from outside obstetrical clinic.

#### Management after delivery and childbirth

The initial medical care is provided by the neonatologist clinic (neonatal intensive care unit [NICU]) for airway management, monitoring, implementation of nasogastric tube (NGT), and information to cleft center.

#### Initial procedure from cleft center

- Taking an impression of the maxilla.
- Fabrication of the plate (prototype) in the laboratory.
- Adjustment and insertion of the plate within 24 hours via endoscope in a transdisciplinary approach (pediatric anesthetist, neonatologist, otolaryngologist).

#### After insertion of the PEBP

- Monitoring of vital parameters, analgesia.
- Regular check whether the plate is correctly fixed, suction of saliva.
- Observing changes of facial profile, body posture, visibility of the tongue tip for 24 to 48 hours.
- Second endoscopic control if signs of desaturation or anxiety are present over 24 hours.
- If the newborn shows no desaturation and breaths calmly for at least 24 hours, the child is transferred from NICU to intermediate care.
- Post 2 to 3 days of PEBP insertion, the parents are trained in handling the plate.

#### Feeding protocol

- Nutrition counseling starts 24 to 48 hours after insertion of the plate.
- First drinking attempt starts then with finger feeding, to control the tongue condition and safe swallowing mechanism. Then, we start with bottle-feeding using 123 teats (Phillips Avent, Philips GmbH).
- The removal of the NGT depends of the amount of intake.

#### Discharge requirements

- No signs of respiratory distress.
- Parents feeling safe with handling the PEBP.
- After training the parents in PBLS (pediatric basic life support).
- Patients, who are not able to reach their amount of intake (100 mL/kg body weight) for the first few days and were discharged with NGT.

### Description of PEBP

#### Construction and adjustment of the PEBP

- We start with taking the C-silicone (Xantopren, Kulzer GmbH) impressions of the palate of neonates in the intensive care unit.
- C-silicones are most suitable due to their controllable viscosity, accuracy, elasticity, and dimensional stability. Using the impression, a medical engineer, in his laboratory, prepares a plaster model with high precision dental plaster (picodent camtec-rock Type IV, Synthetic Superhard Stone Plaster DIN EN 6873, Dental-Produktions-und Vertriebs-GmbH) to make the device.
- The plates are made of hard acrylic (auto-polymerizing methyl methacrylate, Orthocryl, Dentaurum). A velar extension is made of distinct, colored hard acrylic with an average dimension of 1.5 × 4 cm, which is connected by a U-shaped wire of 0.9 mm diameter (remanium, Dentaurum GmbH & Co. KG) and intermittently with 2-0 silk sutures (Johnson & Johnson).
- Should the wire break, the silk suture secures the extension. The use of wire between the PEBP and velar spur enables 3-dimensional positioning of the spur as well as simultaneous bedside modification and immediate insertion.
- Extraoral hooks are added to the plate to fix it on the forehead (Figure 1A and B).

- The adjustment of the PEBP is carried out in the operation theater with the assistance of a pediatric anesthetist, to secure the airway in supine position during the procedure.
- Patient-specific adjustment of the PEBP is done when the patient is awake. The medical engineer present during the adjustment session adapts the size and position of the spur according to the endoscopic findings. During special cases, like mandibular asymmetry, the medical engineer is also present while impression making to assist with the adjustments required for fabricating the PEBP under special anatomical considerations. During the adjustment, the plate is held in situ with the fingers until the extraoral hooks at the end of the plate are bent individually and fixed on the forehead with ribbons.
- For approximately 2 days, the infants undergo routine suction. The necessary pressure is achieved by bending the wires. This pressure is evaluated with a power meter, which is hooked into the ribbons during the resting position, swallowing, and during head turning, without compromising the laryngeal and hyoid elevation; the average pressure estimated is 4 to 5 N (PCE-FB 50 Power meter, PCE Deutschland GmbH).

With respiratory stability and a relaxed body posture overnight, after adapting the PEBP, no further adjustment is required for 3 months. Usually, a new PEBP is needed after 3 months owing to maxillary growth. As palate closure is usually performed after 6 months, 2 PEBPs are needed for the treatment of RS. An average of 30 minutes is required for adjustment of the PEBP.

## Results (2010-2019)

Between January 2010 and December 2019, we treated 132 infants with PEBP. Among them, 111 (84%) had isolated RS (iRS) and 21 (16%) had syndromic RS (sRS). Tracheostomy was needed in 5 of 21 patients with sRS due to unsuccessful treatment with PEBP.

### Breathing Situation Before PEBP Insertion in iRS Cases (n = 111)

- Postnatal stability in the prone position was noted in 31 patients.
- Desaturation and presternal retractions were seen in 80 patients despite prone positioning. Seventy-seven of these patients needed nasopharyngeal airway (NPA) until PEBP insertion. Fifteen needed additional ventilation assistance with positive airway pressure, 3 infants had to be intubated immediately after birth.

### Endoscopic Findings at the Site of Obstruction

**Figure 2.**
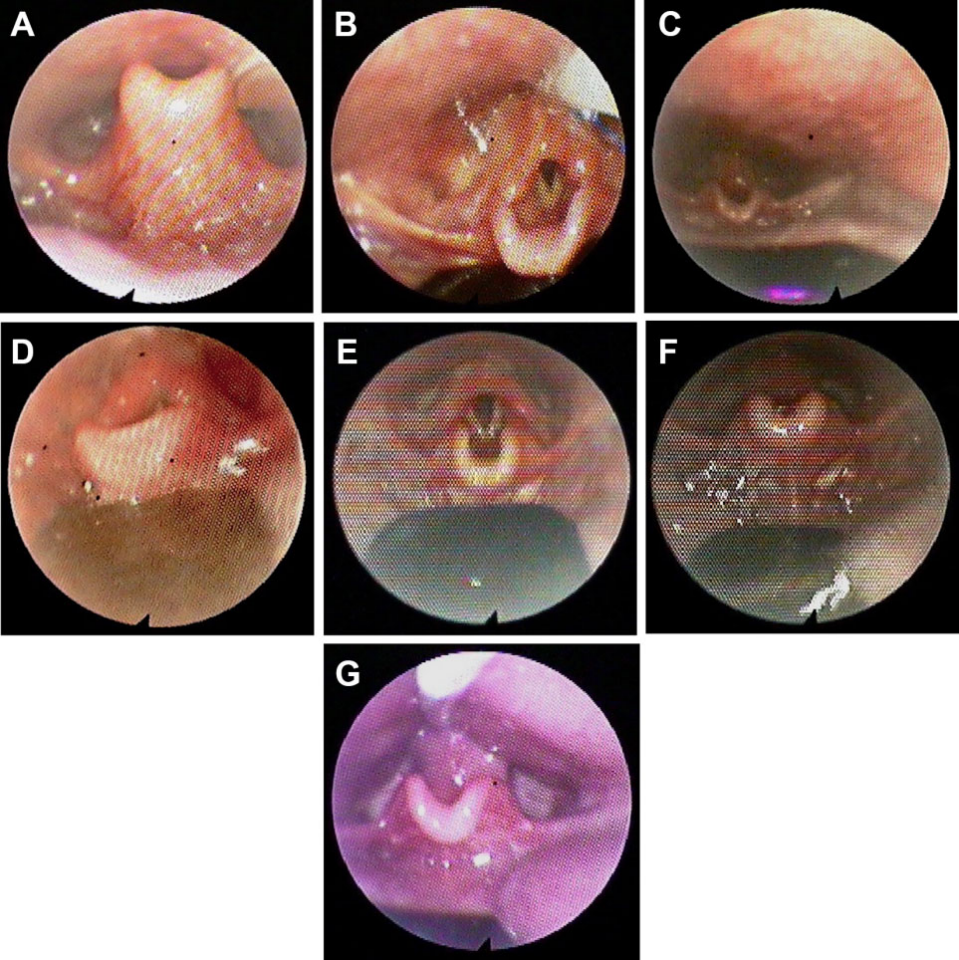
A, Laryngomalacia with instable and dorsally positioned epiglottis to Olney type III. B, Laryngomalacia with pre-epiglottic baton plate (PEBP). Endoscopic view of the epiglottis and the base of the tongue covered by the velar extension of the PEBP (the velar extension is indicated by the arrow). C, A short and wide velar extension (upper airway is kept open). D, A long velar extension reaching the vallecula, with a steeper angle than in Figure 2C (upper airway is kept open). E, Velar extension with a steeper angle than in Figure 2D. F, The cranial and anterior pull of the tongue base. G, Needed widening because of lateral tongue bulge.

*Category I:* Obstruction in the oropharynx: predominantly cranial to the upper rim of the epiglottis.
*Category II:* Obstruction in the oropharynx: the base of the tongue abuts the epiglottis, and the vallecula is not visible.
*Category III:* Obstruction in the oropharynx and hypopharynx: the base of the tongue dislocates the epiglottis posteriorly into the hypopharynx.
*Category IV:* Any of the abovementioned obstructions with a certain type of laryngomalacia with an unstable and dorsally positioned epiglottis (Olney type III; Figure 2A and B) (Olney et al., 1999).

Thereafter, with the PEBP in situ and under endoscopic control, the size, length, and position of the spur as well as the bending of the wires are performed until the glottis appears in full view. This usually causes the tongue and mandible to move forward.

### Technical Design of the Velar Extension Based on Endoscopic Criteria

*For category I:* The velar extension is short and extends maximally until the upper rim of the epiglottis and is as wide as the epiglottis (Figure 2C).
*For category II:* The spur reaches the vallecula with anterior pressure to the base of the tongue and is slightly wider than the epiglottis (Figure 2D).
*For category III:* The spur reaches the vallecula and has a steeper angle to exercise a light anterior pull on the plica glossoepiglottica and plicae pharyngoepiglotticae (Figure 2E).
*For category IV:* The velar extension has a much steeper angle than for patients with category III, which additionally exerts a light cranial pull to straighten the epiglottis (Figure 2F).

In patients with muscular hypotonia, lateral soft tissue bulges of the tongue may restrict the effectiveness of the plate. Therefore, the extension must be broadened until no bulging occurs (Figure 2G). When the spur reaches the vallecula in patients with categories II, III, and IV RS, care must be taken to spare the plica glossoepiglottica and plicae pharyngoepiglottica (Figure 1B). Sparing these structures will avoid pressure sores and impairment of laryngeal elevation while swallowing.

### After Insertion of the PEBP

- Breathing problems diminished in all patients with iRS. Extubation in the 3 children was possible immediately after PEBP insertion in the operation theater. The overall compliance was good; a second adjustment was not necessary.
- Infants were discharged with active surveillance on a home monitor within an average of 8 days after starting PEBP therapy.
- There was no discontinuation or noncompliance rate.

### Feeding Results

- Prior to PEBP insertion, all patients had an NGT.
- With nutrition training and good drinking rhythm, oral intake was possible after 1 to 4 weeks (average 18 days) for all 111 patients with iRS.
- Most infants (n = 105) could consume 90 mL with 123 teats and additional finger feeding, if needed, within 1 hour (average 18 days).
- Seven babies showed delayed weight gain due to long feeding attempts.
- Procedure to gain weight: enriched milk and premature introduction of soft food at the age of 4 months.
- Nasogastric tube longer than 2 weeks after discharge was needed by 4 infants.

### Weight Gain

- Average birth weight was 3.1 kg.
- The mean weight gain: 575 g in the first postnatal month, and 900 g in the first 3 postnatal months; average weight at the age of 6 months was 6000 g.

### Duration of Hospital Stay

Hospital stay was between 4 and 21 days after PEBP insertion, with an average of 8 days.

### After Hospital Discharge

The infants were discharged with a home monitor, followed by intensive recall by pediatricians, nutrition therapists, and subsequent hospital follow-up every 4 weeks.

During our trial and error phase between 2006 and 2010, only patients with clinical signs of UAO despite prone positioning received the PEBP treatment. When the prone positioning was sufficient, and they were drinking on their own, they were discharged without inserting the PEBP, expecting catchup growth. In this period, we had 21 patients where prone positioning was initially successful. Three of the initially sufficient breathing and drinking infants who were discharged without PEBP developed breathing and feeding complications a few weeks later (2 with aspiration), with rehospitalization; 15 developed feeding problems, with failure to thrive. Eventually, of 21 patients, 9 infants remained stable. The fate of these primarily stable patients made us change our protocol. Since then we initiated the insertion of PEBP in all infants with RS, without a wait-and-see in prone position.

Until 2012, we maintained the PEBP in place for 3 months until mouth breathing started and then performed intravelar veloplasty first and hard palate repair 6 months later.

From 2012, owing to a high rate (30%) of difficult intubations at the age of 3 months, we changed our surgical approach from 2 procedures to a single procedure extending the wearing time of the PEBP for better jaw development from 3 to 6 months, with combined closure of the hard and soft palate.

Amelioration of the jaw relation and normalizing the tongue position facilitated the placement of the mouth gag and the surgical approach. By keeping the tongue out of the cleft, PEBP functions such as presurgical orthopedics, allowing the cleft width to decrease. Most of the initially wide U-shaped clefts turned into a V-form. Delay in the timing of the operation due to the width of the cleft was not necessary. These were substantial improvements, noticed incidentally. Post palate-closure surgery, all patients were discharged on postoperative day 5.

## Discussion

This study presents the therapeutic usefulness of a customizable PEBP for treating infants with RS. Our PEBP design, based on the technique described by Pielou in 1967, has the option to bend and adjust the spur immediately. Since 2010, we have been initiating the PEBP therapy immediately in all infants diagnosed with RS, with good outcomes. Our results show that the use of PEBP is effective in treating iRS.

From our perspective, the main difficulty for the implementation of PEBP is the lack of exact description of its adjustment based on multiple anatomical levels. We needed adequate years (13) and patients (132) to substantiate this objective before our first publication.

Prior to PEBP, usual treatment modalities were prone positioning and NPA, with tracheostomy as *ultima ratio*, with unsatisfactory long-term prognosis. Tongue–lip adhesion and MDO were not accepted by the neonatologists in our region. von Bodmann et al. (2003) published the first description of PEBP from Tübingen. Convinced of the effectiveness of this novel nonsurgical procedure, we decided to alter our treatment protocols.

With few resources in the beginning, we eventually modified and designed our own model plate. In collaboration with a team of pediatric anesthesiologist, neonatologist, and pediatric otolaryngologists, who were experienced in the evaluation and treatment of pediatric difficult airways, we started the adjustment stage of PEBP. In addition, the presence of a medical engineer during PEBP was one of the main reasons for our positive outcome.

Our multidisciplinary team constantly supported us during our trial-and-error phase. With experience, we now only require a multidisciplinary team for special patients. Moreover, we have developed a routine that allows us to adjust the PEBP within 30 minutes and enables us to discharge infants within 1 or 2 weeks after PEBP adjustment. During hospital stay, the infants are cared for by the experienced nurses who know how often the child needs to be suctioned, or whether the PEBP is correctly fixed. Pre-epiglottic baton plate cleaning is needed every 2 to 3 days, and nurses teach the parents how to take out, replace, and fix the PEBP correctly.

Before the discharge, the parents receive counseling on feeding issues by a nutritionist and a speech therapist specializing in treating cleft patients. As PEBP therapy is a dynamically adaptable procedure, experienced and interdisciplinary support will make the therapy increasingly comfortable. In this way, the birth of a child with RS is no longer a challenge. With increasing success, we wanted to share our experiences on PEBP with other clinicians.

With PEBP in right position, the parents do not have to worry about the child’s holding, sleeping, and drinking positions. The handling is as simple as an orthodontic plate treatment during childhood. The infant does not need prone positioning or NPA over months, which can be more stressful than a well-accepted plate. Compared to PEBP, surgical interventions are riskier, cost intensive, and require longer hospital stay with additional supportive care (Skirko et al., 2020).

The main advantages of PEBP are immediate and nonsurgical treatment of UAO via correct placement of the tongue, and correctly securing the laryngeal elevation, which subsequently provides a full view of the glottis, without the need for general anesthesia. Moreover, the therapeutic results can be validated via endoscopy, and immediate bedside modification is possible. We are convinced that if properly designed, the PEBP is superior to the other currently available options for managing UAO. At best, PEBP will help clinically convert an infant with iRS into an infant with an isolated cleft palate. We also believe that earlier the PEBP insertion, the better is the acceptance. Due to this reason, we do not practice active surveillance any longer.

To date, we have successfully treated all our patients with iRS. However, in syndromic cases and in patients with associated malformations, for example, neurological problems, hypotonia, choanal stenosis, the treatment with PEBP was not always effective. In one patient, the wire connecting the PEBP broke and the extension was swallowed. It was then removed endoscopically without complications. Therefore, to prevent this risk, we now secure the baton with a 2-0 silk suture in between the wire loop.

We do not perform polysomnography (PSG) routinely in our institution as requested by the Tübinger group. We did not consider it mandatory as long as routine pediatric evaluations of the children with PEBP showed normal development. In selected cases, especially in sRS, we require PSG before soft/hard palate closure after taking out the PEBP for 2 weeks to prevent perioperative risks. When sleep apnea is diagnosed, the wearing time of PEBP is prolonged for a minimum of 2 months, along with delayed surgery.

## Conclusion

We are convinced that the treatment of postnatal UAO is the main but not the only advantage of PEBP. We believe that PEBP helps improve the jaw relation and the tongue position, which make swallowing and drinking possible and reduces anesthesia risks. Maintaining the tongue position away from the cleft reduces the cleft width, which facilitates surgery. All these are preconditions for normal speech and general development, which we intend to confirm in further studies.
